# Extracellular microRNAs in Relation to Weight Loss—A Systematic Review and Meta-Analysis

**DOI:** 10.3390/ncrna9050053

**Published:** 2023-09-14

**Authors:** Camilla H. B. Veie, Isabella M. T. Nielsen, Nanna L. S. Frisk, Louise T. Dalgaard

**Affiliations:** Department of Science and Environment, Roskilde University, Universitetsvej 1, 4000 Roskilde, Denmarknlsn@ruc.dk (N.L.S.F.)

**Keywords:** non-invasive diagnostics, biomarker, liquid biopsy, extracellular RNA, non-coding RNA, microRNA, metabolic syndrome, obesity, weight loss, diet, bariatric surgery, body weight homeostasis

## Abstract

Obesity is an important risk factor for cardiovascular disease and type 2 diabetes mellitus. Even a modest weight loss of 5–15% improves metabolic health, but circulating markers to indicate weight loss efficiency are lacking. MicroRNAs, small non-coding post-transcriptional regulators of gene expression, are secreted from tissues into the circulation and may be potential biomarkers for metabolic health. However, it is not known which specific microRNA species are reproducibly changed in levels by weight loss. In this study, we performed a systematic review and meta-analysis to investigate the microRNAs associated with weight loss by comparing baseline to follow-up levels following intervention-driven weight loss. This systematic review was performed according to the PRISMA guidelines with searches in PubMed and SCOPUS. The primary search resulted in a total of 697 articles, which were screened according to the prior established inclusion and exclusion criteria. Following the screening of articles, the review was based on the inclusion of 27 full-text articles, which were evaluated for quality and the risk of bias. We performed systematic data extraction, whereafter the relative values for miRNAs were calculated. A meta-analysis was performed for the miRNA species investigated in three or more studies: miR-26a, miR-126, and miR-223 were overall significantly increased following weight loss, while miR-142 was significantly decreased after weight loss. miR-221, miR-140, miR-122, and miR-146 were not significantly changed by intervention-driven weight loss. These results indicate that few miRNAs are significantly changed during weight loss.

## 1. Introduction

Throughout recent decades, the prevalence of obesity has grown to become a global health concern [1]. Obesity is a major contributor to the global burden of chronic diseases and cardiometabolic abnormalities, including insulin resistance, β-cell dysfunction, heart diseases, and reduced fertility [2]. The main treatment strategy is to achieve weight loss through lifestyle changes, to reduce energy intake, and to enhance physical activity, resulting in increased energy expenditure [1]. The current guidelines recommend losing 5% to 15% of your body weight in order to improve metabolic function and health outcomes [3].

MicroRNAs (miRNAs) are noncoding RNAs that post-transcriptionally regulate gene expression. This means that they can be key regulators of metabolic homeostasis, and they are involved in regulation of many metabolic diseases, including obesity and the metabolic comorbidities [4]. Intracellularly, the primary role of an miRNA is to downregulate the expression of target genes by interacting with the 3′ untranslated region (3′ UTR) of the target messenger RNA (mRNA) [5]. miRNAs are also released into the circulation from cells encased in extracellular vesicles (EVs) or bound to RNA-binding proteins; these also serve as a promising biomarker type and have been detected in almost all body fluids [6]. More than 2500 miRNAs are identified in the human genome, where they regulate various protein-coding genes [5]. Multiple miRNAs have been quantified in different studies of obese patients to identify whether the expression of certain miRNAs change during intervention-driven weight loss. The use of miRNAs as biomarkers in circulation provides an approach for the early identification of a patient’s risk of developing comorbidities and for observing how well a patient benefits from weight loss [7].

Multiple studies have investigated the circulating levels of specific miRNA species in relation to obesity and comorbidities (reviewed in [8,9,10,11]). It has become apparent that the level of some miRNAs could be affected by intervention-driven weight loss [7,12]. However, studies often carry a low number of subjects, which leads to a risk of bias in reporting and low confidence in the validity of findings. Therefore, the overall purpose of this systematic review using PRISMA guidelines [13,14] is to summarize and integrate previous findings and provide an overview of all the available literature on circulating miRNA changes following intervention-driven weight loss in relation to obesity.

## 2. Results

### 2.1. Data Inclusion

**Figure 1:**
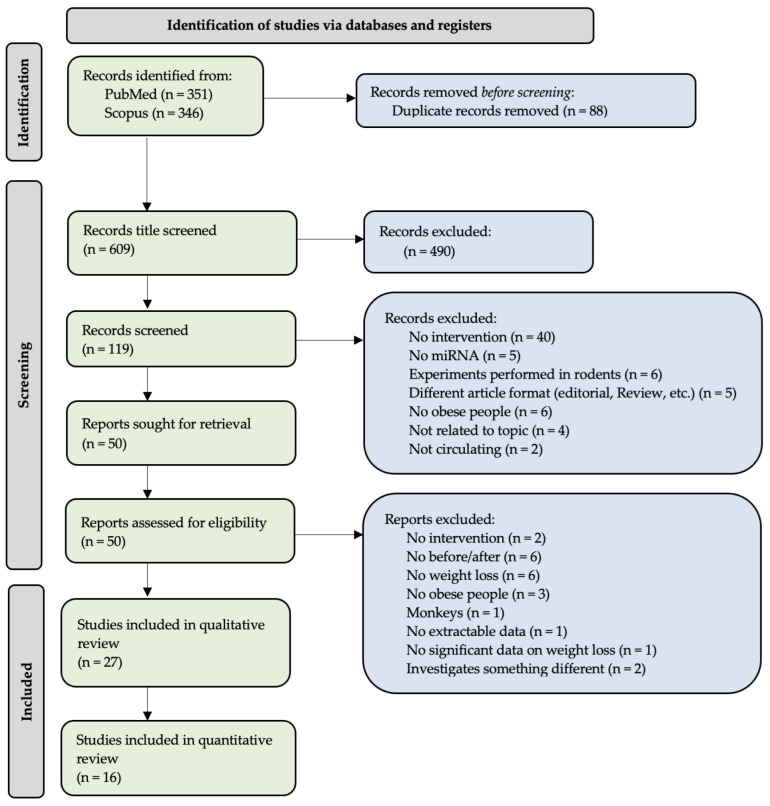
PRISMA flow diagram illustrating the search and inclusion numbers of the systematic review. It resulted in 27 unique articles being included in the systematic review (qualitative review), of which 16 could be included in the meta-analysis (quantitative review).

The inclusion or exclusion of the articles identified during first search was determined using the PRISMA guidelines [13,14]. Additionally, the layout of the systematic review as well as the abstract followed the PRISMA article and abstract checklist, respectively, and can be found in Supplementary Materials Tables S1 and S2. For the PRISMA flow diagram of study inclusion and exclusion (Figure 1): 697 studies were identified through literature searches, 88 studies were duplicates, and 609 studies were title screened for relevance, leaving 119 for abstract screening, which was carried out by two individuals individually, and in cases of disagreement, a third person was the final decision maker. In total, 50 publications were full-text read (Figure 1). We were able to include 16 studies in the meta-analysis (Table 1) and an additional 11 articles in the qualitative part of the systematic review (Table 2). This gives a final number of 27 original articles on which the systematic review and meta-analysis was based (Figure 1) (Supplementary Materials File S3).

### 2.2. Estimation of Study Quality

**Figure 2:**
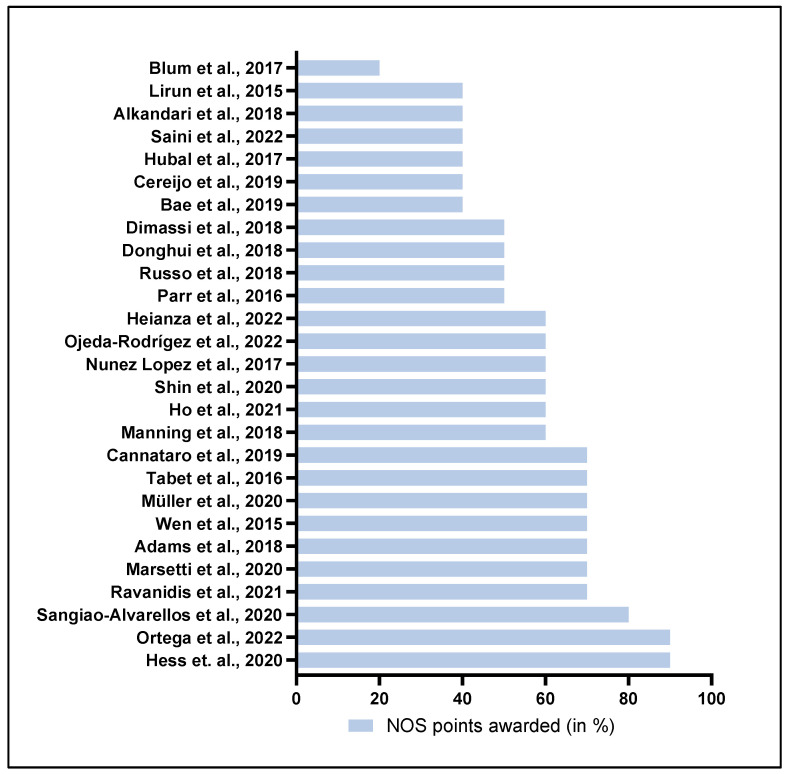
Quality evaluation of the 27 included studies according to the NOS scale. The green scale (left side) corresponds to the percentage of NOS scale points fulfilled, while the blue scale (right side) corresponds to the percentage of NOS scale points not fulfilled. References for the studies: Ortega et al. (2022) [7], Hess et al. (2020) [12], Sangiao-Alvarellos et al. (2020) [38], Cannataro et al. (2019) [16], Tabet et al. (2016) [39], Müller et al. (2020) [36], Wen et al. (2015) [28], Adams et al. (2018) [29], Marsetti et al. (2020) [22], Ravanidis et al. (2021) [25], Heianza et al. (2022) [33], Ojeda-Rodrígez et al. (2022) [23], Nunez Lopez et al. (2017) [20], Shin et al. (2020) [27], Ho et al. (2021) [19], Manning et al. (2018) [21], Dimassi et al. (2018) [17], Donghui et al. (2018) [18], Russo et al. (2018) [26], Parr et al. (2016) [24], Lirun et al. (2015) [35], Alkandari et al. (2018) [30], Saini et al. (2022) [37], Hubal et al. (2017) [34], Cereijo et al. (2019) [32], Bae et al. (2019) [31], and Blum et al. (2017) [15].

The 27 included studies were evaluated using the NOS (Supplementary Materials Fils S3). The NOS is a scale for assessing the quality of non-randomized studies in meta-analyses, giving a possible maximal score of ten stars. The 27 studies were scored on three criteria: selection, comparability, and methods. The criteria are listed in Supplementary Materials File S3, while the NOS evaluation score for each of the 27 studies is displayed in Figure 2, which visualizes the sum of points using a bar plot indicating the combined quality of the study. The diagnosis of obesity is determined by a BMI > 25, following the national guidelines from the WHO [3]. All the studies included had intervention-driven weight loss, with measurements of changes in circulating miRNAs.

### 2.3. Patient Populations in the Included Studies

The studies were analyzed for differences between their study populations. The majority of the studies included both sexes [12,15,16,19,20,22,24,25,26,30,31,32,35,36,37,38], although six articles only investigated the effect of weight loss in women [17,21,23,27,29,34] and four investigated the effects in male subject groups [7,18,33,39]. Only two articles specified the ethnicity of the included participants: African-American women [34] and Caucasian males [7]. Any other definition of ethnicity would be made by the authors based on the last names of any author or co-author as well as the research location. Moreover, there are differences in their intervention approaches; ten studies investigated the effects of dietary changes alone [12,16,21,22,27,29,33,36,39], nine articles investigated the effect of surgery-induced weight loss [15,19,20,30,31,32,34,35,38], and one article investigated the effect of the combination of surgery and diet [7]. Furthermore, two studies investigated the effects of exercise [17,26], whilst four combined the effects of diet and exercise [18,23,25,28], and lastly, one article investigated the effects of a fasting regimen [37]. All studies investigated the effect of the intervention in participants with a BMI > 28 kg/m^2^.

### 2.4. Evaluation of Pre-Analytical Factors

**Figure 3:**
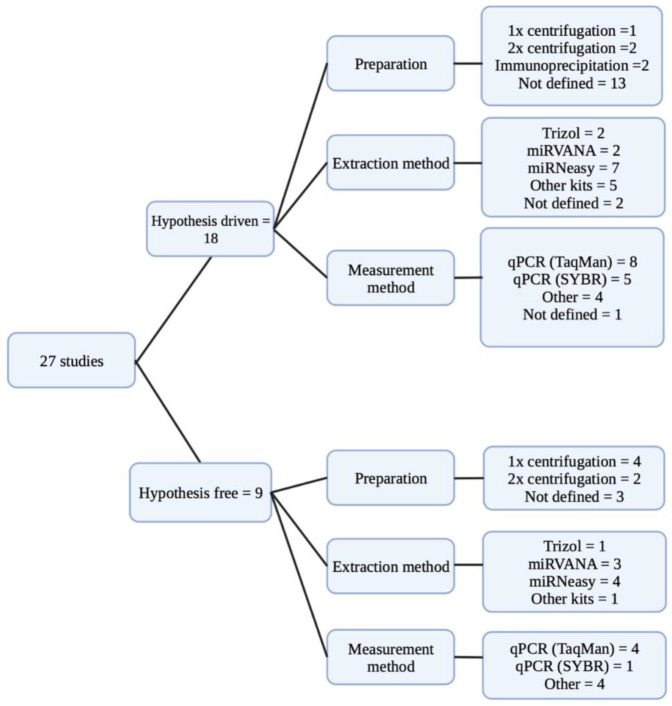
Summary of the pre-analytical factors in the 27 included studies. The underlying data for the figure are available in Supplementary Materials File S3.

The majority of studies investigated miRNAs in plasma [7,17,20,21,23,24,25,26,27,28,30,34,35,36]; the rest were distributed as (41%) investigating serum [12,15,16,18,19,29,31,32,37,38,39] or whole blood (7%) [22,33]. Moreover, 18.5% of the studies used a single-step centrifugation process for plasma or serum separation [16,22,28,35,37], while 15% used a two-step centrifugation [7,17,24,31]. Furthermore, 7.5% used immunoprecipitation [19,39], while 59% of the studies did not provide information about the separation process [12,15,18,20,21,23,25,26,27,29,30,32,33,34,35,38] (Figure 3, Supplementary Materials File S3).

For RNA extraction, the studies used different commercially branded reagents such as Trizol (11%) [12,22,27] or kits such as as miRVANA (18.5%) [7,30,32,34,35] or miRNeasy (41%) [15,16,19,20,21,23,24,31,36,38,39]. Twenty-two percent used other kits [17,18,25,26,28,37], while 7.5 percent did not define the RNA isolation procedure [29,33]. Sixty-seven percent of the studies were hypothesis-driven candidate miRNA studies, whereas thirty-three percent of the studies were hypothesis-free. The measurement methods included Taqman qPCR [7,12,17,18,19,21,26,31,32,38,39], SYBR green qPCR [15,23,27,28,30,36], or other approaches such as NanoString Technology, TaqMan miRNA arraycards, Illumina’s miRNA-seq technology, miScript miRNA PCR array, flowcytometry, Seramir Exosome RNA amplification kit, Affymetrix GeneChip array, unspecified NGS [16,20,22,24,25,29,34,35,37], respectively, or not defined [33].

### 2.5. miRNA Diversity across the Included Studies

**Figure 4:**
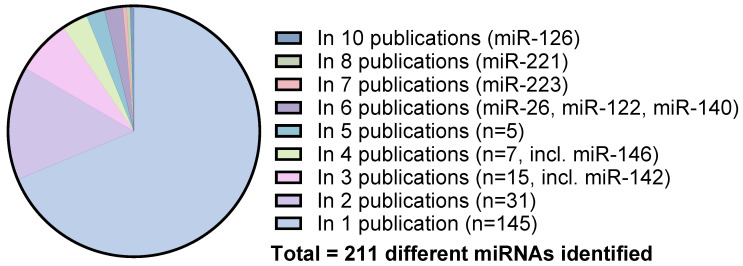
Pie chart overview of the miRNA diversity across the articles found in the systematic review. In total, 211 miRNAs were identified across the studies, of which 32 miRNAs were found in three or more articles; the mentioned 8 miRNAs are the ones included in the meta-analyses.

Across the included 27 articles, 211 miRNAs were identified. The majority (*n* = 176) of the miRNAs were only investigated in one or two studies (Figure 4), which automatically excluded them for further analyses, although, with additional studies on these miRNAs, they could still be shown to have a change in level following weight loss. In total, 34 miRNAs were identified in three or more articles, 15 miRNAs in three articles (miR-193a [12,21,25], miR-34a [12,20,21], miR-143 [12,25,38], miR-19b [25,35,38], miR-22 [15,25,30], **miR-142** [7,25,31], miR-27b [22,25,38], miR-29a [25,30,38], miR-150 [17,25,38], miR-192 [25,30,38], miR-215 [22,25,36], miR-320b [22,30,35], miR-24 [19,35], miR-25 [27,35,36], and miR-30e [15,16,30]); 7 miRNAs were investigated in four different articles (**miR-146** [17,25,26,35], miR-93 [25,30,35,36], miR-222 [12,19,38,39], miR-375 [22,25,33,38], miR-191 [29,31,35,38], miR-15a [20,22,30,31], and miR-92a [29,30,32,35]); 5 miRNAs were each investigated in five different articles (miR-21 [12,15,17,30,38], miR-148b [12,16,30,36,38], miR-486 [15,25,35,36,38], let-7b [29,30,35,36,38], and **miR-106b** [20,29,30,35,36]); some had been investigated in six articles, and this included 5 different miRNAs (**miR-122** [7,12,15,20,25,38], miR-16 [7,12,30,34,35,38], **miR-140** [7,12,31,36,38], **miR-26a** [16,25,27,35,36,38], and miR-320a [15,17,21,22,30,35]); 2 miRNAs had been investigated in seven and eight articles (**miR-223** [12,17,19,24,28,38,39] and **miR-221** [7,12,20,23,24,25,35,38]), respectively); lastly, there was 1 miRNA that was investigated in 9 individual studies (**miR-126**) [12,18,19,21,22,25,26,27,39]) (Figure 4). The miRNAs highlighted in bold are the ones for which it was possible to conduct a quantitative meta-analysis. The remaining miRNAs not highlighted did not present with sufficient extractable data to carry out the meta-analysis. A complete list of the extracted miRNA data can be found in the Supplementary Materials File S3.

### 2.6. Meta-Analysis of the Identified miRNAs

**Figure 5:**
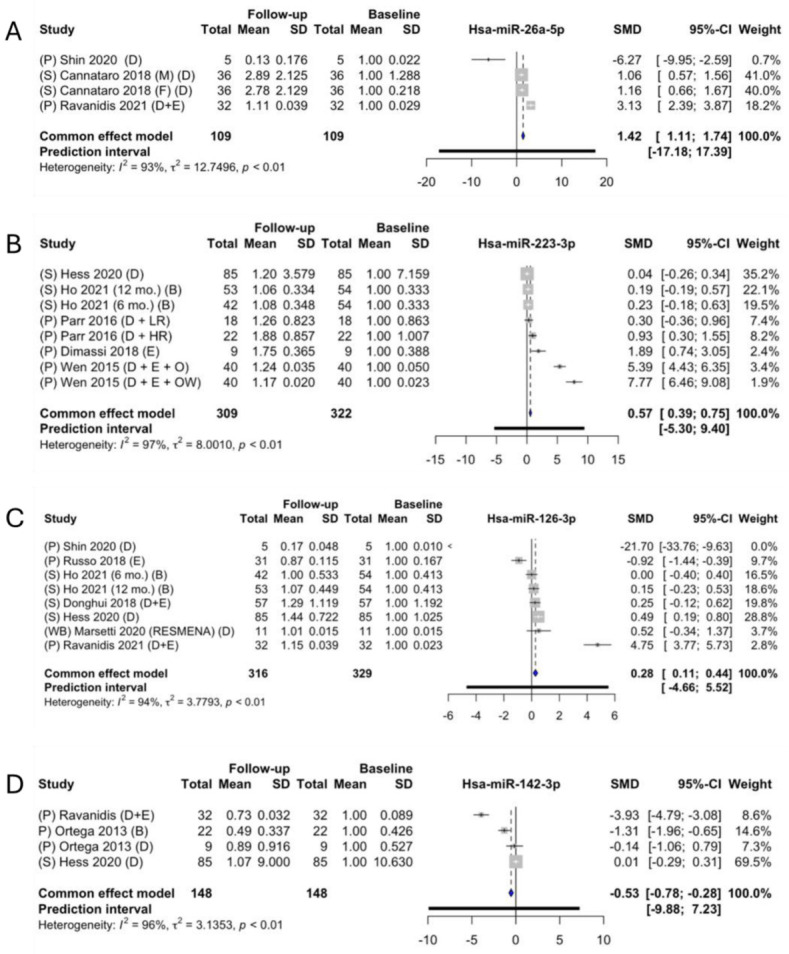
Fixed-effect meta-analyses of miR-26a-5p (**A**), -223-3p (**B**), -126-3p (**C**), and -142-3p (**D**). CI; confidence interval, SD; standard deviation, SMD; standardized mean difference, S; serum, P; plasma, D; diet, M; male, F; female, E; exercise, B; bariatric surgery, O; obese, OW; overweight, RESMENA; specific diet in study. The size of the grey squares indicate the weight of individual studies, while the blue diamond indicates the SMD for the fixed-effects estimate.

**Figure 6:**
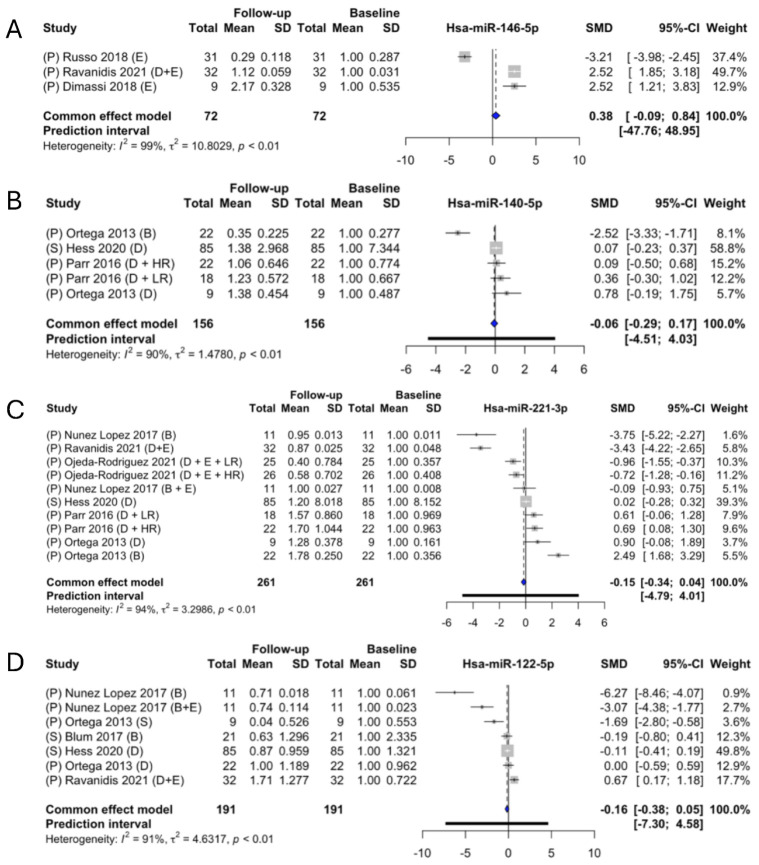
Fixed-effect meta-analyses of miR-146-5p (**A**), -140-5p (**B**), -221-3p (**C**), and -122-5p (**D**). CI; confidence interval, SD; standard deviation, SMD; standard mean difference, S; serum, P; plasma, D; diet, E; exercise, B; bariatric surgery, LR; low responder, HR; high responder, O; obese, OW; overweight, RESMENA; Metabolic Syndrome Reduction in the Navarra diet. The size of the grey squares indicate the weight of individual studies, while the blue diamond indicates the SMD for the fixed-effects estimate.

**Figure 7:**
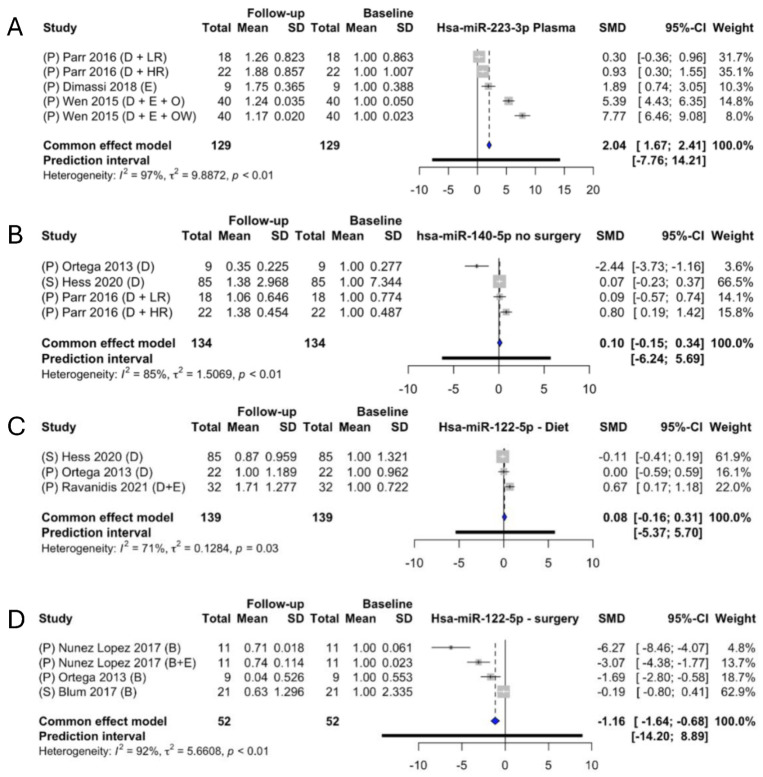
Subgroup fixed-effect meta-analyses of plasma miR-223-3p (**A**), diet miR-140-5p (**B**), diet miR-122-5p (**C**), and surgery miR-122-5p (**D**). CI; confidence interval, SD; standard deviation, SMD; standard mean difference, S; serum, P; plasma, D; diet, E; exercise, B; bariatric surgery, LR; low responder, HR; high responder, O; obese, OW; overweight. The size of the grey squares indicate the weight of individual studies, while the blue diamond indicates the SMD for the fixed-effects estimate.

Eight forest plots were generated, as well as four subgroup analyses (Figure 5, Figure 6 and Figure 7). Three miRNAs were overall significantly upregulated following a weight-loss intervention (miR-26a, miR-223, and miR-126), while one miRNA was found to be significantly downregulated (miR-142) (Figure 5).

miR-26a had the highest-identified fold change (2.42, *p* < 0.05) between the baseline and follow up measurements (SMD: 1.42; 95% CI: [1.11; 1:74]) (Figure 5A). miR-26a was investigated in three of the original studies [16,25,27]. There is significant heterogeneity between the studies (*I*^2^ of 93%, τ^2^ = 12.756, *p* < 0.01), with a prediction interval from −17.18 to 17.39. In the meta-analysis, Shin et al. had a much lower SMD compared with the other two studies. Interestingly, Shin et al. [27] only investigated miRNA expression changes in women, whereas Cannataro et al. and Ravanidis et al. both investigated mixed study populations [16,25].

miR-223 had a with a significant fold change of 1.57 (SMD: 0.57; 95% CI: [0.39; 0.75]) (Figure 5B). For miR-223, four studies presented data that were extractable for the meta-analysis [12,17,24,28]. There was significant heterogeneity between the studies, (*I*^2^ of 97%, τ^2^ = 8.00, *p* < 0.01) with a prediction interval from −6.60 to 11.97. All the studies except for one investigated the changes in mixed-sex groups; the last one included only women [17].

Wen et al. chose to divide their study population into overweight and obese based on the participants’ BMI; the highest SMD was found in their overweight subgroup [28]. Interestingly, the studies investigating the change in serum [12,19] had a much lower SMD than the studies investigating plasma [17,24,28]. Therefore, a subgroup analysis was conducted (Figure 6) to identify the overall regulation of levels of miR-223 only in plasma. From the subgroup analysis of only the studies investigating plasma, the overall SMD increased to 3.04 (SMD: 2.04; CI: [1.67; 2.41]) (Figure 7A).

miR-126 had the lowest fold change of 1.28, although this was significant (SMD: 0.28; 95% CI: [0.11; 0.44]) (Figure 5C). Seven individual studies [12,18,19,22,25,26,27] presented data that could enter the meta-analysis. However, the heterogeneity was significant (*I*^2^ of 93%, τ^2^ = 2.99, *p* < 0.01), with a prediction interval ranging from −3.91 to 4.79. Interestingly, Shin et al. observed a downregulation of miR-126 following weight loss, whereas Ravanidis et al. found that it upregulated. A noticeable difference between the two is that Shin et al. investigated the difference in weight loss in women achieved by following a Korean diet that is high in vegetables and lower in fat; conversely, Ravanidis et al. followed participants of mixed sexes who were fasting with a daily intake of 250 kcal consisting of orange juice, honey, and soup.

Lastly, miR-142 presented a significant fold change of –1.53 (SMD: −0.53; 95% CI; [−0.78; −0.28]) (Figure 5D). Three articles investigated the change in miR-142 [7,12,25], one of which presented data from two subgroups [7]. There was a significant heterogeneity between the studies (*I*^2^ of 96%, τ^2^ = 3.14, *p* < 0.01), with a prediction interval ranging from −9.88 to 7.23. There was one study [25] with a slightly more downregulated SMD than the others, which accounted for 8.6% of the overall weight of the meta-analysis. Interestingly, Ravanidis et al. was also the study in which their time frame is lowest, with 10 ± 3 days [25]; the other two were 12 weeks and 12 months for Hess et al. and Ortega et al., respectively [7,12].

miR-146 had a fold change of 1.38, although not significant, (SMD: 0.38; 95% CI: [−0.09; 0.84]) (Figure 6A). Through the systematic review, three individual articles presenting data that can be used for the meta-analysis were identified [17,25,26]. Furthermore, there was a significant heterogeneity (*I*^2^ of 99%, τ^2^ = 10.80, *p* < 0.01) between studies. One study [26] indicated a downregulation, whereas the two others indicated an upregulation with the same SMD. One study [17] investigated the level of change in women with a mean age of 21 years; on the contrary, the other two [25,26] investigated it in mixed cohorts of 22 males (M)/10 females (F) and 16F/15M, respectively, with a mean age in the mid-50s. The sample sizes of these meta-analyses were quite small: one study only investigated the effect in a sample size of nine [17], whereas the other two studies both included thirty+ participants [25,26].

Overall, miR-140 was not found to be regulated by weight loss (SMD: −0.06; 95% CI: [−0.29; 0.17) (Figure 6B). Three individual studies presented data necessary for the meta-analysis [7,12,24]. Two of the articles presented two subgroups each [7,24]. There was a significant heterogeneity between the studies included (*I*^2^ of 90%, τ^2^ = 1.48, *p* < 0.01), with a prediction interval ranging from −4.51 to 4.03. Parr et al. presented two subgroups, in one of which the participants were defined as high responders (HRs) and low responders (LRs) [24]. Noteworthy is that one of the subgroups in Ortega et al. is the only study that investigated the expression change in a surgery-induced participant group. A subgroup analysis was made to remove the surgery group. The fold change was then −1.10 (*p* > 0.05) (SMD: −0.10; 95% CI; [−0.15;0.34]) (Figure 7B), yet still with heterogeneity (*I*^2^ of 85%, τ^2^ = 1.5069, *p* < 0.01). There was no overall significant difference when the surgery-induced weight loss group was removed, indicating that it was not the intervention method that caused the differences in the findings.

miR-221 followed, with a fold change of –1.15 (*p* > 0.05) (SMD: −0.15; 95% CI; [−0.34; 0.04]) (Figure 6C). Six studies presented the necessary data to conduct the meta-analysis of miR-221 [7,12,20,23,24,25]. There was a significant heterogeneity between the studies (*I*^2^ of 94%, τ^2^ = 3.2986, *p* < 0.01), as evident from the prediction interval between −4.79 and 4.01.

miR-122 had an overall identified fold change of –1.16 (*p* > 0.05) (SMD: −0.16; 95% CI; [−0.38; 0.05]) (Figure 6D). In addition to this, the meta-analysis had significant heterogeneity (*I*^2^ of 91%, τ^2^ = 4.63, *p* < 0.01), with a prediction interval ranging from −7.3 to 4.58. Five articles [7,12,15,20,25] presented the necessary data for inclusion in the meta-analysis. It should be noted that in this analysis, there are different types of interventions, even within the articles, which explain the separate entries from the same study. Nunez Lopez et al. investigated the change in obese people who underwent RYGB but had two different subgroups: one with an exercise program and one without [20]. They observed a higher decrease in the abundance of miR-122 in the group with no exercise program when comparing the two subgroups. Ortega et al. investigated the difference in miRNA expression in two cohort groups, diet-induced weight loss and bariatric-surgery-induced weight loss [7], and they observed a bigger decrease in the expression of miR-122 in their surgery cohort. Interestingly, the studies that investigated the change in expression of miR-122 in dietary-induced weight loss [7,12,25] observed much less of a change than those who investigated the change after surgery-induced weight loss [7,15,20]. Due to the different types of interventions, two subgroup analyses were conducted. A subgroup analysis was made to investigate the effect of diet intervention (Figure 7C), which identified a fold change of –1.08 (SMD: −0.08; 95% CI; −0.16; 0.31]); although this was not significant as *p* > 0.05, it presented significant heterogeneity (*I*^2^ of 91%, τ^2^ = 4.63, *p* < 0.01). The other subgroup analysis was made based on intervention via surgery and gave a significant fold change of –2.16 (SMD: −1.16; 95% CI; [−1.64; −0.68]) (Figure 7D), indicating that miR-122 is significantly downregulated following a surgery-induced weight loss, although the test subject group was quite small, containing only 52 participants.

## 3. Discussion

It has become apparent that the level of specific circulating miRNA species could be affected by weight loss. As organs, tissues, and cells affected by excess weight release miRNAs into circulation, they could possibly be novel biomarkers of the lack of metabolic health in obese patients or predictors of the response to weight loss. Therefore, it is relevant to gather studies on miRNA investigations to research if miRNAs could be a biomarker by determining whether the expression is altered due to an intervention-driven weight loss.

With the aim of gathering the currently available knowledge of circulating miRNAs in relation to controlled interventions for weight loss, 27 studies were included in the current analysis. After data extraction, 16 of the studies were eligible for the meta-analysis. In total, 211 miRNAs were investigated in the 27 included studies, and of those, 34 miRNAs were investigated in three or more of the studies. Eight of the thirty-four miRNAs had extractable data and could be investigated in the meta-analysis to determine if the miRNA level was altered due to an intervention-driven weight loss. Four were identified as upregulated (**miR-26a-5p**, **miR-223-3p**, miR-146-5p, **miR-126-3p**), and four were identified as downregulated (miR-140-5p, miR-221-3p, miR-122-5p, **miR-142-3p**). A subgroup analysis was performed on three of the miRNAs (**miR-223-3p**, miR-140-5p, and **miR-122-5p**), with bold indicating the significant findings.

miR-26a-5p was found to be significantly upregulated after an intervention-driven weight loss (Figure 5A). Multiple studies have reported that miR-26a-5p plays a crucial role by acting as a tumor suppressor in the liver, an important organ involved in maintaining lipid and glucose homeostasis [40]. Furthermore, several studies have determined that there is a downregulation of miR-26a-5p in overweight human and obese mouse models and that target genes are involved in fatty acid synthesis, gluconeogenesis, and insulin signaling [41,42].

miR-223-3p, which is increased after weight loss intervention, is characterized as a part of the hematopoietic system, where it is expressed in the myeloid compartment. The levels of miR-223 are altered in cardiometabolic disease, regulate hepatic cholesterol metabolism, and are very abundant in circulation [11,28,39,43,44,45,46,47,48]. One study demonstrated an increased expression in the myeloid-derived cells and demonstrated that miR-223 controls granulocyte and macrophage number and function as well [49]. Macrophage activation plays a crucial role in regulating the adipose tissue and is a major contributor to the pathogenesis of obesity-associated diseases, such as cardiovascular diseases [50]. Moreover, there is solid evidence that miR-223 is released from platelets during blood coagulation, resulting in different baseline levels of this miRNA in serum compared with plasma [51,52,53]. Three studies in the meta-analysis investigated the change in serum and five investigated the change in plasma. Therefore, a subgroup analysis was made to test whether the changes in circulating miR-223 levels induced by weight loss could be more measurable in plasma than serum. From the sub-group analysis, the SMD by weight loss is increased in plasma, with a combined fold change of 2.04, supporting the hypothesis that the lower SMD in the first analysis was due to a high baseline abundance of miR-223 in the serum samples and that a subgroup analysis with only plasma samples would lead to an increased degree of regulation of miR-223.

miR-126-3p was found to be significantly upregulated by weight loss. It has been observed that miR-126 has been associated with the development of T2DM. Multiple studies showed the downregulation of miR-126 as an indicator of developing T2DM [48,54]. One study found that there was a downregulation of miR-126 in circulation even before the development of T2DM, which suggests that the level of circulating miR-126 could be used to predict which participants would develop T2DM [54]. The observation of miR-126 being significantly upregulated following intervention-driven weight loss supports the notion of miR-126 being a T2DM biomarker, as weight loss would increase the levels of miR-126, and through weight loss, the participants decrease their risk of developing T2DM.

miR-140-5p and miR-142-3p were both found to be downregulated following intervention-driven weight loss. Studies have observed that the two miRNAs are associated with a promotion of adipogenesis [55]. A downregulation of miR-140-5p and miR-142-3p is in concordance with the fact that overexpression leads to adipogenesis, as weight loss would reduce adipose tissue size.

miR-221-3p is associated with metabolic diseases, specifically adipocyte differentiation, and the expression of miR-221-3p is found to be directly associated with obesity. There is a direct relationship with the expression of miR-221-3p in adipose tissue and BMI, as there is a higher expression of miR-221-3p in adipose tissue in obese people compared with normal-weight subjects [7]. However, the meta-analysis was not able to demonstrate a significant regulation of miR-221 in circulation following weight loss.

The liver-enriched miRNA miR-122-5p is expressed almost solely in hepatocytes and reaches almost 70% of the total miRNA population in the adult liver. This highly suggests that miR-122 plays an essential role in hepatocyte differentiation and its regulation. Furthermore, miR-122 plays a role in cholesterol biosynthetic pathways and fatty acid metabolism and is found to be overexpressed in fatty liver patients and increased in the patient’s blood [8,56]. It has been shown that there is an association between an increased risk of insulin resistance and the circulating levels of miR-122 [57,58] and that there is an increase in miR-122 expression in obese patients when compared to healthy controls [57].

The findings of the meta-analysis of miR-146 could suggest that following weight loss, miR-146 is slightly upregulated. However, this was not found to be significant, and the included studies of the analysis are inconsistent in the sense that they indicate contradicting results. One study [26] found miR-146 to be downregulated, whereas two other studies [17,25] found it to be upregulated. They all investigated the effect of a low-calorie diet on miRNA abundance.

Several limitations are inherent when performing a systematic review and a meta-analysis: Biomarker studies are often affected by publication bias, where insignificant associations are less reported than significant associations are. In this systematic review, we only included published studies, which might potentially lead to the exclusion of studies showing insignificant associations. In order to reduce publication bias, we have included all data, including the Supplementary Materials from the included studies. For the meta-analysis, we included circulating miRNAs, which at least three studies had reported data on. Thus, it is likely that the miRNAs not included in the meta-analysis could still have a relation to weight loss.

Moreover, for most of the investigated miRNAs in the meta-analysis, we identified significant heterogeneity among the studies. One explanation for the observed heterogeneity could be how the study populations vary by ethnicity, sample material, sex, age, and time of intervention, as those factors are likely to influence the measured levels of miRNA. Furthermore, a selection bias may also be present, as only nine of the studies were based on hypothesis-free approaches while eighteen were hypothesis-driven.

While the research field of circulating miRNAs as biomarkers is immature and still developing, our meta-analysis showed that the circulating levels of several miRNAs are significantly altered in obese patients due to intervention-driven weight loss. Our findings were obtained despite the challenge that the meta-analysis included a relatively low number of studies investigating the same miRNAs, as many miRNAs were only represented once (Figure 4). Thus, additional miRNAs might be associated with obesity and weight loss than those investigated in the current meta-analysis, as this review focused on miRNAs investigated in three or more studies. Another limitation was that not all the identified studies could be included in the meta-analysis as the data were not extractable, which potentially limited the strength of this meta-analysis. Moreover, as more studies of miRNAs as biomarkers are published, it will be important in future studies to assess diagnostic accuracy over multiple studies using summary receiver operator characteristics analyses.

## 4. Materials and Methods

Predating the search for articles, a Prospero protocol (CRD42022362905) was submitted (7 October 2022) [59]. PRISMA 2020 guidelines were followed in determining the included studies [13,14] and performing the systematic review. Furthermore, the PRISMA checklist and PRISMA abstract checklists have been followed to ensure the full inclusion of necessary information and can be found in Supplementary Materials Tables S1 and S2.

### 4.1. Data Extraction

The search engines PubMed (https://pubmed.ncbi.nlm.nih.gov/ (accessed on 8 August 2022)) and Scopus (https://www.scopus.com/search/form.uri?display=basic#basic (accessed on 8 August 2022)) were used to identify 697 articles through systematic searches using the search string (weight loss OR diet) AND (miRNA OR miR) NOT murine AND circulating NOT review NOT Meta-analysis). After duplicate removal, 609 articles remained to be title screened, and following title screening, 119 remained to be abstract screened. Abstract screening was carried out by two individuals (IMTN and CHBV), and in cases of disagreement, a third person was the final decision maker. Abstract screening yielded 50 studies for full-text reading. We were able to include 27 articles in the qualitative part of the systematic review and 16 in the meta-analysis part. We included studies that had at least one overweight group with measurements of miRNA levels following intervention-driven weight loss. Intervention was defined as “an outside instigator promoting changes in weight of the participants” but was not dependent on method. The methods could be physical or dietary interventions as well as bariatric surgery. The included studies had to have a quantitative measurement of circulating miRNA.

Extensive data extraction of all the articles was performed to give an overview of the included articles. Publication information, miRNAs identified, sample sizes, intervention method, intervention time frame, sample material, normalization method, as well as specific raw data were included in the data extraction, which can be found in Supplementary Materials File S3. When data were not written in tables, we used the online freeware WebPlot digitizer version 4.6 (https://apps.automeris.io/wpd/ accessed on 19 September 2022) to extract quantitative miRNA levels and the degree of variation from graphs. For comparability of the included articles for each measurement, we calculated the levels of miRNA relative to the level of measurement at baseline, which was set to one. Furthermore, we calculated the standard deviation (SD) relative to the level of miRNA at each time point. Data presented in a logarithmic scale were recalculated to arithmetic scale. Formulas used for this as well as for the reverse calculation of SEM can be found in Supplementary Materials File S3. All available data for each study were extracted, including any data found in Supplementary Materials, thus trying to reduce the risk of biases.

### 4.2. Study Quality Estimations

We used an adjusted Newcastle–Ottawa Scale (NOS) [60] to estimate the quality of the included studies. The adjusted NOS can be found in Supplementary Materials File S3.

### 4.3. Statistics and Futher Data Analysis

The heterogeneity of the included studies was evaluated by investigating the effects of the differences in sample material, intervention method, timeframe of intervention, ethnicity, and measurement methods through a qualitative analysis. The quantitative analysis was conducted by performing a meta-analysis on levels of specific miRNAs, which had been reported on in at least three separate studies and for which extractable quantitative data were available. The measurements of baseline miRNA level and their degree of variation as well as the follow-up measurements and their degree of variation were considered extractable. The R packages meta, metagear, metafor, readxl, tidyverse, and devtools were used for performing the meta-analyses with fixed-effects models. Funnel plots were used to estimate biases, and any outlier found was inspected manually. All statistical evaluations were performed in R-studio software (Version: 2022.07.2). For the organization of data, Excel 365 (Version: 16.75.2) was used. Plotting was carried out using Graphpad Prism vs. 10. Significance was determined at *p*-values < 0.05.

## 5. Conclusions

In conclusion, we observed changes in the levels of specific miRNAs following an intervention-driven weight loss, in particular miR-26a-5p, -223-3p, -126-3p, and -142-3p. Although some population samples were quite small, there were differences in the separation, extraction, and quantification methods, as well as differences in the study populations. This limits the strength of the findings, and therefore, more research is needed to confirm the findings within this study.

## Figures and Tables

**Table 1:** List of the 16 studies included in the meta-analysis part of the systematic review.

Study	Title	Reference	Meta-Analysis
Blum et al. (2017)	Elevated Levels of miR-122 in Serum May Contribute to Improved Endothelial Function and Lower Oncologic Risk Following Bariatric Surgery	[15]	√
Cannataro et al. (2019)	Ketogenic Diet Acts on Body Remodeling and MicroRNAs Expression Profile	[16]	√
Dimassi et al. (2018)	Microparticle miRNAs as Biomarkers of Vascular Function and Inflammation Response to Aerobic Exercise in Obesity?	[17]	√
Donghui et al. (2019)	Improvement of microvascular endothelial dysfunction induced by exercise T and diet is associated with microRNA-126 in obese adolescents	[18]	√
Hess et al. (2020)	Levels of Circulating miR-122 are Associated with Weight Loss and Metabolic Syndrome	[12]	√
Ho et al. (2021)	High density lipoprotein-associated miRNA is increased following Roux-en-Y gastric bypass surgery for severe obesity	[19]	√
Nunez Lopez et al. (2017)	Gastric bypass surgery with exercise alters plasma microRNAs that predict improvements in cardiometabolic risk	[20]	√
Manning et al. (2019)	Acute Weight Loss Restores Dysregulated Circulating MicroRNAs in Individuals Who Are Obese	[21]	√
Marsetti et al. (2021)	Changes in miRNA expression with two weight-loss dietary strategies in a population with metabolic syndrome	[22]	√
Ojeda-Rodríguez et al. (2022)	Circulating miRNAs in girls with abdominal obesity: miR-221-3p as a biomarker of response to weight loss interventions	[23]	√
Ortega et al. (2013)	Targeting the Circulating MicroRNA Signature of Obesity	[7]	√
Parr et al. (2016)	Circulating MicroRNA Responses between ‘High’ and ‘Low’ Responders to a 16-Wk Diet and Exercise Weight Loss Intervention	[24]	√
Ravanidis et al. (2021)	Fasting-mediated metabolic and toxicity reprogramming impacts circulating microRNA levels in humans	[25]	√
Russo et al. (2018)	Physical Activity Modulates the Overexpression of the Inflammatory miR-146a-5p in Obese Patients	[26]	√
Shin et al. (2020)	A Traditional Korean Diet Alters the Expression of Circulating MicroRNAs Linked to Diabetes Mellitus in a Pilot Trial	[27]	√
Wen et al. (2015)	Circulating microRNA-223 as a potential biomarker for obesity	[28]	√

**Table 2:** List of the 11 studies only included in the qualitative systematic review.

Study	Title	Reference
Adams et al. (2018)	Exercise and weight loss interventions and miRNA expression in women with breast cancer	[29]
Alkandari et al. (2018)	Improved physiology and metabolic flux after Roux-en-Y gastric bypass is associated with temporal changes in the circulating microRNAome: a longitudinal study in humans	[30]
Bae et al. (2019)	Bariatric Surgery Alters microRNA Content of Circulating Exosomes in Patients with Obesity	[31]
Cereijo et al. (2020)	Elevated Levels of Circulating miR-92a Are Associated with Impaired Glucose Homeostasis in Patients with Obesity and Correlate with Metabolic Status After Bariatric Surgery	[32]
Heianza et al. (2022)	Changes in Circulating miR-375-3p and Improvements in Visceral and Hepatic Fat Contents in Response to Lifestyle Interventions: The CENTRAL Trial	[33]
Hubal et al. (2017)	Circulating adipocyte-derived exosomal microRNAs associated with decreased insulin resistance after gastric bypass	[34]
Lirun et al. (2015)	A Pilot Study: The Effect of Roux-en-Y Gastric Bypass on the Serum MicroRNAs of the Type 2 Diabetes Patient	[35]
Müller et al. (2020)	SNP dependent modulation of circulating miRNAs from the miR25/93/106 T cluster in patients undergoing weight loss	[36]
Saini et al. (2022)	Time-Restricted Eating Regimen Differentially Affects Circulatory miRNA Expression in Older Overweight Adults	[37]
Sangiao-Alvarellos et al. (2020)	Metabolic recovery after weight loss surgery is reflected in serum microRNAs	[38]
Tabet et al. (2016)	High-Density Lipoprotein-Associated miR-223 Is Altered after Diet-Induced Weight Loss in Overweight and Obese Males	[39]

## Data Availability

All data are available in Supplementary Materials File S3.

## Supplementary Materials

The following are available online at https://www.mdpi.com/article/10.3390/ncrna9050053/s1, Supplementary Materials Table S1: PRISMA Checklist, Table S2: PRISMA Abstract checklist, File S3: Data extraction sheet and NOS evaluations.

## References

[1] World Health Organization (2021). Obesity and Overweight: Fact Sheets. https://www.who.int/news-room/fact-sheets/detail/obesity-and-overweight.

[2] Afshin A., Forouzanfar M.H., Reitsma M.B., Sur P., Estep K., Lee A., Marczak L., Mokdad A.H., Moradi-Lakeh M., Naghavi M. (2017). Health Effects of Overweight and Obesity in 195 Countries over 25 Years. N. Engl. J. Med..

[3] World Health Organization (2000). Obesity: Preventing and managing the global epidemic. Report of a WHO consultation. World Health Organ. Tech. Rep. Ser..

[4] Price N.L., Ramirez C.M., Fernandez-Hernando C. (2014). Relevance of microRNA in metabolic diseases. Crit. Rev. Clin. Lab. Sci..

[5] Vienberg S., Geiger J., Madsen S., Dalgaard L.T. (2017). MicroRNAs in metabolism. Acta Physiol..

[6] Chen X., Ba Y., Ma L., Cai X., Yin Y., Wang K., Guo J., Zhang Y., Chen J., Guo X. (2008). Characterization of microRNAs in serum: A novel class of biomarkers for diagnosis of cancer and other diseases. Cell Res..

[7] Ortega F.J., Mercader J.M., Catalán V., Moreno-Navarrete J.M., Pueyo N., Sabater M., Gómez-Ambrosi J., Anglada R., Fernández-Formoso J.A., Ricart W. (2013). Targeting the circulating microRNA signature of obesity. Clin. Chem..

[8] Atic A.I., Thiele M., Munk A., Dalgaard L.T. (2023). Circulating microRNAs associated with non-alcoholic fatty liver disease. Am. J. Physiol. Cell Physiol..

[9] Brandao B.B., Lino M., Kahn C.R. (2022). Extracellular miRNAs as mediators of obesity-associated disease. J. Physiol..

[10] Santos D., Carvalho E. (2022). Adipose-related microRNAs as modulators of the cardiovascular system: The role of epicardial adipose tissue. J. Physiol..

[11] Dinesen S., El-Faitarouni A., Dalgaard L.T. (2023). Circulating microRNAs associated with gestational diabetes mellitus: Useful biomarkers?. J. Endocrinol..

[12] Hess A.L., Larsen L.H., Udesen P.B., Sanz Y., Larsen T.M., Dalgaard L.T. (2020). Levels of Circulating miR-122 are Associated with Weight Loss and Metabolic Syndrome. Obesity.

[13] Page M.J., McKenzie J.E., Bossuyt P.M., Boutron I., Hoffmann T.C., Mulrow C.D., Shamseer L., Tetzlaff J.M., Akl E.A., Brennan S.E. (2021). The PRISMA 2020 statement: An updated guideline for reporting systematic reviews. BMJ.

[14] Page M.J., Moher D., Bossuyt P.M., Boutron I., Hoffmann T.C., Mulrow C.D., Shamseer L., Tetzlaff J.M., Akl E.A., Brennan S.E. (2021). PRISMA 2020 explanation and elaboration: Updated guidance and exemplars for reporting systematic reviews. BMJ.

[15] Blum A., Yehuda H., Geron N., Meerson A. (2017). Elevated Levels of miR-122 in Serum May Contribute to Improved Endothelial Function and Lower Oncologic Risk Following Bariatric Surgery. Isr. Med. Assoc. J..

[16] Cannataro R., Perri M., Gallelli L., Caroleo M.C., De Sarro G., Cione E. (2019). Ketogenic Diet Acts on Body Remodeling and MicroRNAs Expression Profile. Microrna.

[17] Dimassi S., Karkeni E., Laurant P., Tabka Z., Landrier J.F., Riva C. (2018). Microparticle miRNAs as Biomarkers of Vascular Function and Inflammation Response to Aerobic Exercise in Obesity?. Obesity.

[18] Donghui T., Shuang B., Xulong L., Meng Y., Yujing G., Yujie H., Juan L., Dongsheng Y. (2019). Improvement of microvascular endothelial dysfunction induced by exercise and diet is associated with microRNA-126 in obese adolescents. Microvasc. Res..

[19] Ho J.H., Ong K.L., Cuesta Torres L.F., Liu Y., Adam S., Iqbal Z., Dhage S., Ammori B.J., Syed A.A., Rye K.A. (2021). High density lipoprotein-associated miRNA is increased following Roux-en-Y gastric bypass surgery for severe obesity. J. Lipid Res..

[20] Nunez Lopez Y.O., Coen P.M., Goodpaster B.H., Seyhan A.A. (2017). Gastric bypass surgery with exercise alters plasma microRNAs that predict improvements in cardiometabolic risk. Int. J. Obes..

[21] Manning P., Munasinghe P.E., Bellae Papannarao J., Gray A.R., Sutherland W., Katare R. (2019). Acute Weight Loss Restores Dysregulated Circulating MicroRNAs in Individuals Who Are Obese. J. Clin. Endocrinol. Metab..

[22] Marsetti P.S., Milagro F.I., Zulet M.A., Martinez J.A., Lorente-Cebrian S. (2021). Changes in miRNA expression with two weight-loss dietary strategies in a population with metabolic syndrome. Nutrition.

[23] Ojeda-Rodriguez A., Assmann T.S., Alonso-Pedrero L., Azcona-Sanjulian M.C., Milagro F.I., Marti A. (2022). Circulating miRNAs in girls with abdominal obesity: miR-221-3p as a biomarker of response to weight loss interventions. Pediatr. Obes..

[24] Parr E.B., Camera D.M., Burke L.M., Phillips S.M., Coffey V.G., Hawley J.A. (2016). Circulating MicroRNA Responses between ‘High’ and ‘Low’ Responders to a 16-Wk Diet and Exercise Weight Loss Intervention. PLoS ONE.

[25] Ravanidis S., Grundler F., de Toledo F.W., Dimitriou E., Tekos F., Skaperda Z., Kouretas D., Doxakis E. (2021). Fasting-mediated metabolic and toxicity reprogramming impacts circulating microRNA levels in humans. Food Chem. Toxicol..

[26] Russo A., Bartolini D., Mensa E., Torquato P., Albertini M.C., Olivieri F., Testa R., Rossi S., Piroddi M., Cruciani G. (2018). Physical Activity Modulates the Overexpression of the Inflammatory miR-146a-5p in Obese Patients. IUBMB Life.

[27] Shin P.K., Kim M.S., Park S.J., Kwon D.Y., Kim M.J., Yang H.J., Kim S.H., Kim K., Chun S., Lee H.J. (2020). A Traditional Korean Diet Alters the Expression of Circulating MicroRNAs Linked to Diabetes Mellitus in a Pilot Trial. Nutrients.

[28] Wen D., Qiao P., Wang L. (2015). Circulating microRNA-223 as a potential biomarker for obesity. Obes. Res. Clin. Pract..

[29] Adams B.D., Arem H., Hubal M.J., Cartmel B., Li F., Harrigan M., Sanft T., Cheng C.J., Pusztai L., Irwin M.L. (2018). Exercise and weight loss interventions and miRNA expression in women with breast cancer. Breast Cancer Res. Treat..

[30] Alkandari A., Ashrafian H., Sathyapalan T., Sedman P., Darzi A., Holmes E., Athanasiou T., Atkin S.L., Gooderham N.J. (2018). Improved physiology and metabolic flux after Roux-en-Y gastric bypass is associated with temporal changes in the circulating microRNAome: A longitudinal study in humans. BMC Obes..

[31] Bae Y.U., Kim Y., Lee H., Kim H., Jeon J.S., Noh H., Han D.C., Ryu S., Kwon S.H. (2019). Bariatric Surgery Alters microRNA Content of Circulating Exosomes in Patients with Obesity. Obesity.

[32] Cereijo R., Taxeras S.D., Piquer-Garcia I., Pellitero S., Martinez E., Tarasco J., Moreno P., Balibrea J., Puig-Domingo M., Jimenez-Pavon D. (2020). Elevated Levels of Circulating miR-92a Are Associated with Impaired Glucose Homeostasis in Patients with Obesity and Correlate with Metabolic Status After Bariatric Surgery. Obes. Surg..

[33] Heianza Y., Krohn K., Yaskolka Meir A., Wang X., Ziesche S., Ceglarek U., Bluher M., Keller M., Kovacs P., Shai I. (2022). Changes in Circulating miR-375-3p and Improvements in Visceral and Hepatic Fat Contents in Response to Lifestyle Interventions: The CENTRAL Trial. Diabetes Care.

[34] Hubal M.J., Nadler E.P., Ferrante S.C., Barberio M.D., Suh J.H., Wang J., Dohm G.L., Pories W.J., Mietus-Snyder M., Freishtat R.J. (2017). Circulating adipocyte-derived exosomal MicroRNAs associated with decreased insulin resistance after gastric bypass. Obesity.

[35] Lirun K., Sewe M., Yong W. (2015). A Pilot Study: The Effect of Roux-en-Y Gastric Bypass on the Serum MicroRNAs of the Type 2 Diabetes Patient. Obes. Surg..

[36] Muller S., Wallner S., Schmitz G., Loew T., Stempfl T., Mohle C., Strack C., Sag S., Baessler A., Fischer M. (2020). SNP dependent modulation of circulating miRNAs from the miR25/93/106 cluster in patients undergoing weight loss. Gene.

[37] Saini S.K., Singh A., Saini M., Gonzalez-Freire M., Leeuwenburgh C., Anton S.D. (2022). Time-Restricted Eating Regimen Differentially Affects Circulatory miRNA Expression in Older Overweight Adults. Nutrients.

[38] Sangiao-Alvarellos S., Theofilatos K., Barwari T., Gutmann C., Takov K., Singh B., Juiz-Valina P., Varela-Rodriguez B.M., Outeirino-Blanco E., Duregotti E. (2020). Metabolic recovery after weight loss surgery is reflected in serum microRNAs. BMJ Open Diabetes Res. Care.

[39] Tabet F., Cuesta Torres L.F., Ong K.L., Shrestha S., Choteau S.A., Barter P.J., Clifton P., Rye K.A. (2016). High-Density Lipoprotein-Associated miR-223 Is Altered after Diet-Induced Weight Loss in Overweight and Obese Males. PLoS ONE.

[40] Xu J., An P., Winkler C.A., Yu Y. (2020). Dysregulated microRNAs in Hepatitis B Virus-Related Hepatocellular Carcinoma: Potential as Biomarkers and Therapeutic Targets. Front. Oncol..

[41] Wroblewski A., Strycharz J., Oszajca K., Czarny P., Swiderska E., Matyjas T., Zieleniak A., Rucinska M., Pomorski L., Drzewoski J. (2023). Dysregulation of Inflammation, Oxidative Stress, and Glucose Metabolism-Related Genes and miRNAs in Visceral Adipose Tissue of Women with Type 2 Diabetes Mellitus. Med. Sci. Monit..

[42] Xu S., Wang Y., Li Z., Hua Q., Jiang M., Fan X. (2022). LncRNA GAS5 Knockdown Mitigates Hepatic Lipid Accumulation via Regulating MiR-26a-5p/PDE4B to Activate cAMP/CREB Pathway. Front. Endocrinol..

[43] Sanchez-Ceinos J., Rangel-Zuniga O.A., Clemente-Postigo M., Podadera-Herreros A., Camargo A., Alcala-Diaz J.F., Guzman-Ruiz R., Lopez-Miranda J., Malagon M.M. (2021). miR-223-3p as a potential biomarker and player for adipose tissue dysfunction preceding type 2 diabetes onset. Mol. Ther. Nucleic Acids.

[44] Udesen P.B., Glintborg D., Sorensen A.E., Svendsen R., Nielsen N.L.S., Wissing M.L.M., Andersen M.S., Englund A.L.M., Dalgaard L.T. (2020). Metformin decreases miR-122, miR-223 and miR-29a in women with polycystic ovary syndrome. Endocr. Connect..

[45] Vickers K.C., Landstreet S.R., Levin M.G., Shoucri B.M., Toth C.L., Taylor R.C., Palmisano B.T., Tabet F., Cui H.L., Rye K.A. (2014). MicroRNA-223 coordinates cholesterol homeostasis. Proc. Natl. Acad. Sci. USA.

[46] Yoffe L., Polsky A., Gilam A., Raff C., Mecacci F., Ognibene A., Crispi F., Gratacos E., Kanety H., Mazaki-Tovi S. (2019). Early diagnosis of gestational diabetes mellitus using circulating microRNAs. Eur. J. Endocrinol..

[47] Zampetaki A., Kiechl S., Drozdov I., Willeit P., Mayr U., Prokopi M., Mayr A., Weger S., Oberhollenzer F., Bonora E. (2010). Plasma microRNA profiling reveals loss of endothelial miR-126 and other microRNAs in type 2 diabetes. Circ. Res..

[48] Zhu H., Leung S.W. (2015). Identification of microRNA biomarkers in type 2 diabetes: A meta-analysis of controlled profiling studies. Diabetologia.

[49] Leierseder S., Petzold T., Zhang L., Loyer X., Massberg S., Engelhardt S. (2013). MiR-223 is dispensable for platelet production and function in mice. Thromb. Haemost..

[50] Castoldi A., Naffah de Souza C., Camara N.O., Moraes-Vieira P.M. (2015). The Macrophage Switch in Obesity Development. Front. Immunol..

[51] Krammer T.L., Mayr M., Hackl M. (2020). microRNAs as promising biomarkers of platelet activity in antiplatelet therapy monitoring. Int. J. Mol. Sci..

[52] Parker W.A.E., Schulte C., Barwari T., Phoenix F., Pearson S.M., Mayr M., Grant P.J., Storey R.F., Ajjan R.A. (2020). Aspirin, clopidogrel and prasugrel monotherapy in patients with type 2 diabetes mellitus: A double-blind randomised controlled trial of the effects on thrombotic markers and microRNA levels. Cardiovasc. Diabetol..

[53] Willeit P., Zampetaki A., Dudek K., Kaudewitz D., King A., Kirkby N.S., Crosby-Nwaobi R., Prokopi M., Drozdov I., Langley S.R. (2013). Circulating microRNAs as novel biomarkers for platelet activation. Circ. Res..

[54] Zhang T., Li L., Shang Q., Lv C., Wang C., Su B. (2015). Circulating miR-126 is a potential biomarker to predict the onset of type 2 diabetes mellitus in susceptible individuals. Biochem. Biophys. Res. Commun..

[55] Liu Y., Wu W., Zhou L., Cheng L., Miao C. (2018). MicroRNA-142a-3p promotes the differentiation of 3T3-L1 preadipocytes by targeting high-mobility group AT-hook 1. Int. J. Clin. Exp. Pathol..

[56] Wen J., Friedman J.R. (2012). miR-122 regulates hepatic lipid metabolism and tumor suppression. J. Clin. Investig..

[57] Wang R., Hong J., Cao Y., Shi J., Gu W., Ning G., Zhang Y., Wang W. (2015). Elevated circulating microRNA-122 is associated with obesity and insulin resistance in young adults. Eur. J. Endocrinol..

[58] Willeit P., Skroblin P., Moschen A.R., Yin X., Kaudewitz D., Zampetaki A., Barwari T., Whitehead M., Ramirez C.M., Goedeke L. (2017). Circulating MicroRNA-122 Is Associated With the Risk of New-Onset Metabolic Syndrome and Type 2 Diabetes. Diabetes.

[59] Page M.J., Shamseer L., Tricco A.C. (2018). Registration of systematic reviews in PROSPERO: 30,000 records and counting. Syst. Rev..

[60] Wells G.A., Shea B., O’Connel D., Peterson J., Welch V., Losos M., Tugwell P. (2022). The Newcastle-Ottawa Scale (NOS) for Assessing the Quality of Nonrandomised Studies in Meta-Analyses. https://www.ohri.ca/programs/clinical_epidemiology/oxford.asp.

